# Contrast sensitivity and aspects of binocular vision alteration in school-aged children after head injury

**DOI:** 10.22336/rjo.2023.63

**Published:** 2023

**Authors:** Victoria Verejan

**Affiliations:** * Department of Ophthalmology, “N. Testemițanu” State University of Medicine and Pharmacy, Chişinău, Republic of Moldova

**Keywords:** contrast sensitivity, binocular vision, head trauma, children

## Abstract

**Aim:** The research aimed to establish whether contrast sensitivity is a reliable method of evaluation in the case of school-aged children after head injury, and also to establish aspects of binocular vision alteration in the acute phase of TBI.

**Materials and methods:** Forty-eight individuals with persisting visual symptoms after brain injury have been examined.

**Results:** The rate of contrast sensitivity was determined to be 61%-100% in the research group in 56,3%-58,3% cases, compared to the control group, in which the prevalence was 93,7%-95,8% cases. Repeated evaluation during 4 months after the head trauma revealed an incidence of 83,3%-89,6% for the research group and 97,9% for the control group in the same 61%-100% interval. Binocular vision proved to be unchanged in 79,17% of patients, being determined as absent only in 4,16% of patients who later presented a secondary divergent strabismus.

**Conclusions:** Contrast sensitivity is an easily performed method for the group of school-aged children after head injury. Although it is often considered by children an interesting game, its results should be taken into consideration while suspecting a traumatic optic neuropathy. Since most of the pediatric patients aged between 7-18 years, show a slight decrease of contrast sensitivity ability after head trauma, this examination should be performed as a part of ophthalmological evaluation in pediatric patients following head injury.

## Introduction

Visual dysfunctions after mild traumatic brain injury (mTBI) can influence all aspects of vision: visual acuity, accommodation, visual field, photosensitivity, color perception, contrast sensitivity, pupillary functions, saccadic movements, and visual memory. Symptoms of alteration may be present because of damage to the afferent, efferent, or intermittent branches of the visual field [**1**-**3**].

Damage to the afferent pathways after mTBI can manifest itself with changes in visual acuity, contrast sensitivity, and color perception. As a rule, these manifestations are bilateral. In case of post-traumatic optic neuropathy or direct injury to the orbit, the symptoms can be unilateral. Direct trauma can be easily diagnosed using standard ophthalmic equipment. In patients with retrobulbar trauma, signs of proptosis, ptosis, and decreased color perception will be suspected, according to the literature [**1**,**2**].

The projection of the visual analyzer on the cerebral map topography is in the area of the occipital lobe in areas 17, 18, and 19 [**4**]. Field 17, the striated area, located in the calcarine fissure, is the primary visual area that has a role in perceiving the shape of objects and light contrast as a distinctive function. Area 17 is surrounded by parastriate area 18, with a role in differentiating moving objects, and peristriate area 19, with a role in differentiating current visual sensations from those previously stored. The central neuron articulates with Meynert’s receptive cortical cells [**5**].

During the first six months of life, rapid anatomical development of the eyeballs and central visual pathways takes place, parallel to the progression of visual functions: visual acuity, contrast sensitivity, and color perception [**6**].

R. Armstrong reports that few studies confirm color perception dysfunction in patients after mTBI. However, there is data in the literature proving that a case-control study conducted on 11 patients after mTBI and 11 control patients would suggest a deficit perception of a primary color [**7**]. Lemke et al. determined that 21% of patients had low contrast sensitivity after mTBI, which resulted in low quality of life [**8**].

Contrast sensitivity of the visual analyzer is considered to be the ability of a person to perceive details of an object at a low contrast level, often regarded as a non-specific test for the assessment of neurological function. The principle of the method consists of presenting a series of images with a decreasing visual contrast and recording the level at which the subject is no longer able to perceive. Several conditions can affect the ability to perceive differences in contrast. These can be deficiencies of some nutritional factors, excessive alcohol consumption, secondary medicinal effects as well as exposure to various biotoxins, venom, fungi, parasite infections, and heavy metal poisoning.

Its utility as a method for evaluating visual function has been demonstrated in cases in which changes in the usual indicators are not determined: visual acuity or visual field. The contrast is created by the difference between the reflected light - the brightness - of two adjacent surfaces. The contrast is usually expressed as a percentage, the fraction being multiplied by 100. If the lowest perceived contrast is 5%, the contrast sensitivity will be expressed as 100/5=20. If the lowest contrast perceived by a person is 0.6%, the contrast sensitivity will be S.C=100/0.6=170. If a person can distinguish details at very low contrast, their contrast sensitivity is high, and vice versa. There are different contrast levels: 100%, 25%, 10%, 5%, 2.5%, 1.25%, 0.6%. Determining contrast sensitivity can be done using letter systems, figures, or grids that are displayed as boards or on video monitors. Testing can be done under photopic, mesopic, and scotopic conditions [**9**]. Contrast sensitivity is the first of the affected visual functions and the last to return to normal in neuro-ophthalmological diseases. The online contrast sensitivity test is identical to the contrast sensitivity test used in neurotoxin research. Its advantage is that it can be easily adapted to the monitor of the gadget to which the patient will be connected [**10**]. The numerical evaluation is individual for each type of test. According to data from the literature, the most common evaluation criterion is the Pelli-Robson score used in the test of the same name, in which the numerical value is rendered logarithmically equal to 2 units for a 100% contrast sensitivity [**11**].

Case-control studies on patients with mild TBI in the subacute stage and practically healthy patients revealed a higher frequency of binocular and motor dysfunctions [**12**]. Disturbance of binocular vision often occurs after TBI, with the onset of a latent phoria or fusion disorder [**13**]. One of the main complaints of the patient is diplopia, which can appear even when he/she is ready to be discharged [**14**]. Patients will report doubled images as being shifted horizontally, vertically, or even diagonally [**15**].

## Materials and methods

The study was carried out through the clinical evaluation of patients aged between 7-18 years, hospitalized in the Department of Neurosurgery of the IMSP Mother and Child Institute. To achieve the research objectives, a cohort study was conducted. The online contrast sensitivity test (VCSTest.com) was used in the study.

*Research inclusion criteria*

L1 batch eligibility criteria:

- School-aged children who presented visual disorders after TBI;

- No organic pathology of the visual analyzer;

- Patients without other associated chronic pathology.

L0 batch eligibility criteria:

- School-aged children who presented visual disorders with no TBI;

- No organic pathology of the visual analyzer;

- Patients without other associated chronic pathology.

The examination of binocular function had to fulfill a group of conditions:

- Fusion capacity was preserved;

- The parallelism of the eyeballs and ocular motility was preserved;

- Visual acuity was not less than 0.3 diopters;

- There were no organic lesions of the eyeball, which might have been accompanied by a decrease in visual acuity.

In the framework of the research, the Lange I and II stereoscopic tests have been used. The latter can be easily used in children, as it involves recognizing pictures on a board. Each of the two panels shows three images that assess the child’s levels of stereoscopic perception. The principles underlying the method are random distribution of points and grids. The image perceived by each eye is separated by a fine system of parallel lines. There are two versions of the test:

- Lange I (cat, car, star), which measures the stereo capacity from 1200 to 550 seconds;

- Lange II (elephant, car, moon), which measures the stereo capacity from 600 to 200 seconds.

The interpretation of the results is done simply. The positive result will be considered in the case of recognition of all images, usually being associated with research movements of the eyeballs. Negative results will be attributed to a lack of recognition of any images. The child will look without interest at the board. Relatively positive result: only one or two objects will be recognized by the patient, but he will continue to search the board to detect new images. The assessment of the test will be based on the number of images recognized by the patient: positive result 3/3 answers; relative positive result 3/3 partially recognized or 2/3 recognized, of which the omitted image shows 550" arc of stereoacuity level. Negative result 3/3 negative responses or 2/3 negative responses in which the recognized image is at the 1200" level of arc [**16**].

The results of the contrast sensitivity evaluation revealed an increased distribution for both eyes in the 61-100% range: respectively for the right eye in 56,3% cases in the research group, versus 95,8% cases - in the control group (x2 = 12.702 a, gl=2, p1 < 0.01) and for the left eye -58,3% cases - in the research group, compared to 93,7% cases - in the control group (x2 = 17.890a, gl= 2, p1 < 0.001) (**Table 1**). The 41-60% range was more frequently determined among the patients of the researched group, respectively for the right eye in 35,4% of cases, compared to 2,1% of cases - in the control group, and for the left eye: in 29,2% of cases among the children of the researched group, versus 2,1% cases - in the control group. Likewise, the contrast sensitivity revealed an interval of 0-40% for the right eye in 8,3% of cases in the research group, compared to 2,1% of cases - in the control group, and for the left eye in 12,5% cases, versus 4,2% cases.

**Table 1:** Contrast sensitivity ability in school-aged children after mTBI

Eye	Values	L1		L0	
		Patients	%	Patients	%
Right	0%-40%	4	8,3	1	2,1
	41%-60%	17	35,4	1	2,1
	61%-100%	27	56,3	46	95,8
x2 = 12,702a, gl=2, p1 < 0,01					
Left	0%-40%	6	12,5	2	4,2
	41%-60%	14	29,2	1	2,1
	61%-100%	28	58,3	45	93,7
x2 = 17,890a, gl=2, p1 < 0,001					

Patients were re-evaluated after 4 months after trauma and the same procedure was undergone. The results of the contrast sensitivity repeated over 4 months revealed an increased distribution for both eyes in the range of 61-100%: respectively for the right eye in 83,3% of cases in the research group, versus 97,9% of cases - in the control group (x2 = 12.702a, gl=2, p1 < 0.01) and for the left eye - 89,6% cases - in the research group, compared to 97,9% cases - in the control group (x2 = 17.890a, gl=2, p1 < 0.01) (**Table 2**). The 41-60% range was determined among the patients of the control group, respectively for the right eye in 2,1% cases, compared to 16,7% cases - in the research group, and for the left eye: 2,1% cases in the control group and 10,4% - in the research group.

**Fig. 1:**
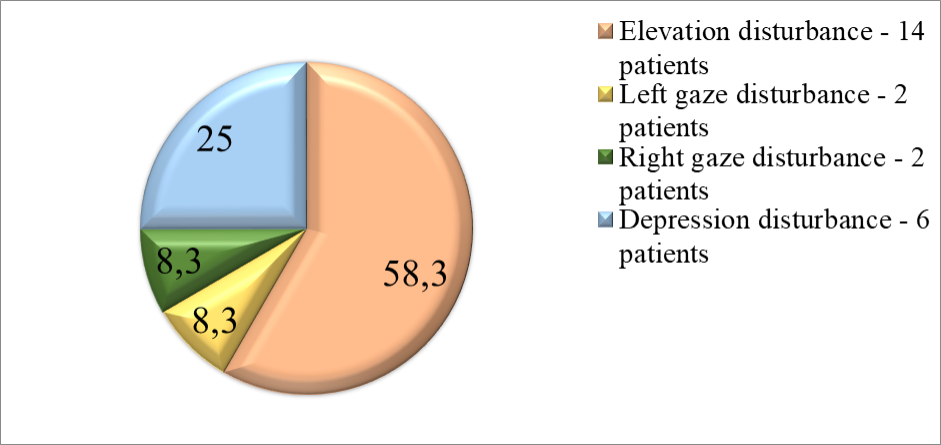
Ocular motility in school-aged patients after mTBI in the acute phase

Ocular motility was tested during the research so that 14 (58,3%) patients presented elevation disturbances, 2 patients (8,3%) - left gaze disturbances, 2 patients (8,3%) - had right gaze disturbances and 6 patients (25%) had depression disorders (**Fig. 1**).

**Table 2:** Contrast sensitivity ability in school-aged children after 4 months of mTBI

Eye	Values	L1		L0	
		Patients	%	Patients	%
Right	0%-40%	0	0	0	0
	41%-60%	8	16,7	1	2,1
	61%-100%	40	83,3	47	97,9
x2 = 12,702a, gl=2, p1 < 0,01					
Left	0%-40%	0	0	0	0
	41%-60%	5	10,4	1	2,1
	61%-100%	43	89,6	47	97,9
x2 = 17,890a, gl=2, p1 < 0,001					

**Fig. 2:**
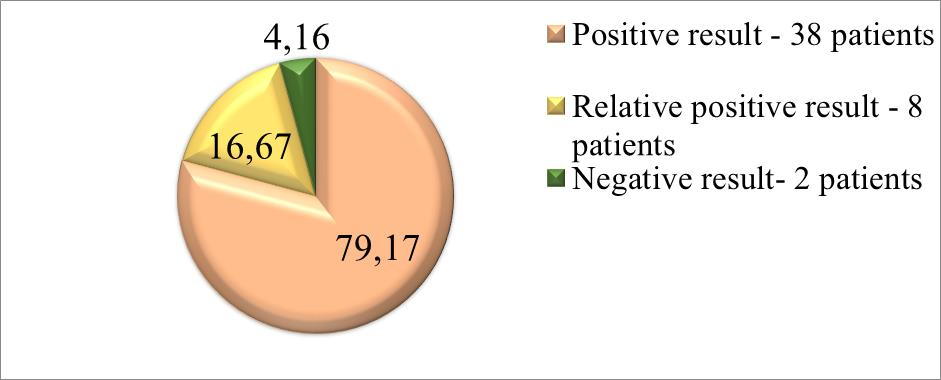
Lange I test results in school-aged patients after mTBI in the acute phase

Examination of binocular function was determined to be positive (recognition of images) in 38 patients, a relatively positive result (perception of images but unable to name them) was determined in 8 patients, and a negative result (non-recognition of images) in the case of 2 patients (**Fig. 2**).

## Discussions

Evaluation of contrast sensitivity revealed changes in the acute phase of mTBI in the case of patients in the research group with an improvement in 4 months after the trauma. Thus, initially, the contrast capacity rate was determined to be 61%-100% for 56,3%-58,3% of cases, compared to the control group with a prevalence of 93,7%-95,8% of cases. Repeated evaluation revealed an incidence of 83,3%-89,6% for the research group and 97,9% for the control group in the same reference interval.

Contrast sensitivity is a function also affected by moderate brain damage. The impact on this function is not a total one, with children being able to distinguish the contrast of bands in the number of approximately 50%-60%, but this could negatively influence their educational performance. What should be mentioned is that investigations related to the evaluation of the visual field and contrast sensitivity could be decisive at the initial stage for the determination of traumatic optic neuropathy. Binocular vision proved to be unchanged in 79,17% of patients, being determined as absent only in the case of 4,16% of patients who later presented secondary divergent strabismus. In the acute stage of the mTBI, contrast sensitivity becomes altered but not as much as to be noticed by the patient himself. This investigation should be included as a part of a complex evaluation in the case of a school-aged child after TBI, but its results should also be correlated to the data provided by procedures such as visual field examination or visual potential evaluation.

As far as binocular alterations are concerned, ocular motility can be present in the acute stage of the brain injury, but they should not be considered as defined, since the results of our research showed that these changes are transient and do not need immediate treatment. Most of the patients showed an elevation disturbance probably due to the onset of secondary cerebral hypertension, but without a significant alteration of binocular ability.

## Conclusions

1. Contrast sensitivity ability becomes altered in school-aged children after mTBI in the immediate acute stage. Our research outlined that 8,3%-12,5% of patients had less than 20% contrast sensitivity and 29,2%-35,4% of patients had contrast sensitivity limited between 21%-60%. After 4 months of head trauma, only 10,4%-16.7% of patients manifested a contrast sensitivity between 21%-60%, most of them having fully reestablished this ability (83,3%-89,6%).

2. Binocular function alteration tested by the Lange I test remained unchanged in school-aged children after mTBI in the immediate acute stage (79,2%). Only patients who showed an exophoria or esophoria had this function severely altered.

3. Ocular motility in school-aged children after mTBI in the immediate acute stage showed an elevation disturbance in 58,3% of cases, probably due to a secondary traumatic onset of cerebral hypertension.

4. Contrast sensitivity evaluation, along with binocular function assessment and ocular motility evaluation should be a part of a complex ophthalmological check in school-aged children after TBI.

**Conflict of Interest Statement**

The author declares no conflict of interest.

**Informed Consent and Human and Animal Rights Statement**

Informed consent was obtained from the parents of the individuals included in this study.

**Authorization for the use of human subjects**

Ethical approval: The research related to human use complied with all relevant national regulations and institutional policies, followed the tenets of the Helsinki Declaration, and was approved by the Research Ethics Board of “N. Testemiţanu” State University of Medicine and Pharmacy, Chişinău, Republic of Moldova on 21/05/2018, No. 63. The trial was the authors’ initiative. The author is independent and takes full responsibility for the integrity of the data and the accuracy of the data analysis.

**Acknowledgments**

None.

**Sources of Funding**

None.

**Disclosures**

None.
